# Prevalence of depression among diabetic patients and its relationship to diabetes self-care activities, disease profile, and social factors in Khartoum State, Sudan: A cross-sectional study

**DOI:** 10.1097/MD.0000000000042350

**Published:** 2025-05-09

**Authors:** Sara Emad, Sara Elawad, Shaima Omer Moahmed Elawad, Ahmed Balla M. Ahmed, Mohamed H. Elbadawi, Leena Khalid, Tibyan Yahya Mohammedelhassan Abdalhamed, Wafa Sosal, Aya M. Haiba

**Affiliations:** a Faculty of Medicine, University of Khartoum, Khartoum, Sudan.

**Keywords:** depression, diabetes mellitus, Patient Health Questionnaire 9, Self-Reported Diabetes Self-Care Activities Questionnaire, Sudan

## Abstract

Depression is common among individuals with diabetes mellitus, yet many cases go undiagnosed. It is linked to poorer treatment outcomes. However, data from developing countries remains limited. This study aimed to assess the prevalence and severity of depression among diabetic patients in Khartoum State. It also examined the association between depression and diabetes self-care activities in addition to other clinical and personal factors. A descriptive cross-sectional study was conducted at 3 diabetic outpatient clinics in Khartoum State. Depression was measured using the Patient Health Questionnaire 9, while diabetes self-management behaviors were evaluated using the Self-Reported Diabetes Self-Care Activities Questionnaire. Clinical and personal characteristics were also collected. Associations between depression and various factors were analyzed using the chi-square test and Fisher’s exact test, with a *P*-value of less than .05 considered statistically significant. A total of 163 participants were included. The overall prevalence of depression among diabetic patients was 86.5%, with the majority experiencing minimal (33.1%) and mild (30.1%) depression. Depression was significantly associated with the type of diabetes medications (*P* = .011) and a family history of psychiatric illness (*P* = .001). Depression was also significantly linked to the loss of a close person (*P*-value = .001) and lack of social support (*P*-value = .018), as well as various aspects of diabetes self-care activities. This study revealed a high prevalence of depression among diabetic patients in Khartoum State. Depression was strongly associated with various diabetes self-care activities as well as other clinical and personal factors. Integrating mental health support into diabetes care programs is essential to improve outcomes. Future population-based studies with more robust methodologies are recommended.

## 1. Introduction

Diabetes mellitus (DM) is a condition in which the body either fails to produce sufficient insulin or does not respond to it effectively, leading to elevated blood sugar (glucose) levels.^[1]^ According to the International Diabetes Federation Diabetes Atlas (2021), 10.5% of adults aged 20 to 79 have diabetes, with nearly half of them unaware that they have the condition.^[2]^ Managing diabetes is challenging and involves optimizing existing treatments to maintain glycemic, blood pressure, and lipid control while minimizing complications. Key challenges include educating patients on self-management, improving adherence to lifestyle and medication plans, reducing resistance to early insulin use, and enhancing healthcare delivery for those with chronic conditions.^[3]^ The demanding lifestyle of diabetes often leads to common psychiatric and psychological comorbidities, which are linked to poorer health outcomes.^[4]^

People with type 1 or type 2 diabetes are at a higher risk of developing depression, anxiety, and eating disorders, with depression rates being twice as high compared to the general population.^[5]^ Major depression is estimated to affect around 12% of diabetic patients (ranging from 8% to 18%), while milder forms of depression or elevated depressive symptoms are reported in 15% to 35% of cases.^[6]^ The presence of depression in diabetic patients worsens the prognosis, reduces the quality of life, and raises the mortality rate.^[7]^ It also raises the risks of microvascular and macrovascular complications, as well as increasing healthcare costs.^[8]^ Research indicates that depression is linked to poor adherence to essential self-care behaviors, such as blood sugar monitoring and maintaining a healthy diet, which, in turn, leads to poor glycemic control and a higher risk of diabetes-related complications.^[9]^

Despite the well-established link between depression and diabetes management, there is limited data specific to Sudan, particularly regarding how depression affects self-care activities among diabetic patients. With the growing burden of diabetes, understanding the prevalence of depression and its impact on disease management is essential for developing effective healthcare interventions tailored to the local population. This study aimed to determine the prevalence of depression among diabetic patients in Khartoum State, the capital of Sudan. It also sought to explore the association between depression and diabetes self-care activities, disease profile, and social factors.

## 2. Methods

### 2.1. Study design and setting

This was a descriptive cross-sectional, multicenter study. It was conducted between December 20, 2020, and February 20, 2021, at 3 diabetic outpatient clinics in Khartoum State.

The first clinic was located at Soba University Hospital, a major tertiary care hospital in southern Khartoum. The second site was the Khartoum University outpatient clinic, which provides primary healthcare services, diagnostic testing, and an ophthalmology unit. The third was the outpatient clinic at General Omar Sawi Medical Complex, a recently established facility under the police healthcare services.

These clinics serve patients from diverse educational and socioeconomic backgrounds, providing a representative sample of the population.

### 2.2. Study population

The study included diabetic patients receiving treatment at the outpatient clinics of Soba University Hospital, Khartoum University clinics, and Omar Sawi Medical Complex during the study period. Patients with a recent diabetes diagnosis (less than 1 year) were excluded.

### 2.3. Sample size and sampling technique

The sample size was determined using the single proportion formula (Cochran equation),^[10]^ with a 95% confidence level, a population proportion of 12%,^[6]^ and a margin of error of 0.05.


$$\begin{array}{l} n=t{}^{2}pq/d{}^{2} \end{array}$$

Where n = sample size, t = standard error corresponding to the 95% confidence level, p = population proportion (prevalence), q = 1 – p, and d = margin of error.

The calculated sample size was 163. Data collection was carried out using nonprobability convenience sampling, where patients attending their scheduled consultations at each clinic were consecutively enrolled until the target sample size was reached. Each clinic operated on specific days of the week.

### 2.4. Data collection tools

Data were collected through patient interviews, with clinical data verified against the patients’ medical records. A structured questionnaire was used, consisting of 5 sections.

The first section gathered information on the sociodemographic characteristics of the patients. The second section covered the diabetes profile and medical history. The third section utilized the Patient Health Questionnaire 9 for the assessment of depression.^[11]^ The Patient Health Questionnaire 9 is a self-reported tool that evaluates depression symptoms, with each of the 9 questions rated on a scale from 0 (not present) to 3 (present most of the time). A score of less than 5 indicates minimal depression, scores between 5 and 9 indicate mild depression, 10 to 14 moderate depression, 15 to 19 moderately severe depression, and 20 to 27 severe depression.^[11,12]^

The fourth section employed the Self-Reported Diabetes Self-Care Activities Questionnaire to assess diabetes self-management behaviors, including diet, exercise, blood glucose testing, and foot care.^[13]^ The results focused on identifying patients who performed these self-care activities infrequently (once a week or less), rather than reporting the average number of days these activities were performed in the previous week.

The fifth section gathered social and individual information. STROBE reporting guidelines for cross-sectional studies were followed to ensure the study was conducted and reported properly.^[14]^

### 2.5. Ethics approval and consent to participate

Ethical approval for this study was obtained in accordance with the Declaration of Helsinki from the University of Khartoum, Faculty of Medicine Ethical Approval Committee. Additional permission was granted by the administration of the clinics involved. Diabetic patients provided written informed consent after the study objectives and data collection procedures were explained in clear, simple terms. No harm was caused to participants, and confidentiality of all patient information was maintained throughout the study.

### 2.6. Data analysis

The collected data were reviewed for completeness before analysis using the Statistical Package for the Social Sciences version 26. Categorical variables were described using frequency tables, while continuous variables were summarized with measures of central tendency. The results were presented in tables, figures, and accompanying comments. The association between depression prevalence and sociodemographic characteristics, medical history, clinical factors, and diabetes self-care activities was assessed using the chi-square test and Fisher’s exact test. Binomial logistic regression was performed for the depression status variable (no depression to mild depression vs. moderate to severe depression). Variables with *P*-value less than .05 were selected to enter the model. A *P*-value of less than .05 was considered statistically significant.

## 3. Results

### 3.1. Sociodemographic characteristics of the study participants

A total of 163 individuals participated in this study, with the majority being female (63.2%, n = 103). The median age of the participants was 51 ± 21 years. Most were married (80.4%), and nearly half (45.5%) had completed primary or secondary education. Almost half of the participants (50.3%) were either unemployed or housewives. Only 36 participants (22.1%) had a good level of income of above 30 thousand SDGs (more than 544.5 U.S. dollars), about 10 times the 2020 minimum wage. None of the participants reported a history of alcohol consumption, and only 3.7% were smokers (Table 1).

**Table 1:** Sociodemographic characteristics of the study participants (N = 163).

Variable	Frequency	Percentage (%)
Age (years) (median ± interquartile range)	51 ± 21	
Gender Male Female	60 103	36.8 63.2
Marital status Married Single Divorced Widow Separate	131 11 1 18 2	80.4 6.7 0.6 11 1.2
Educational level Illiterate Informal education Primary education Secondary education University education Higher education	35 7 36 38 37 10	21.5 4.3 22.1 23.3 22.7 6.1
Occupation Student Governmental employee Private sector employee Freelancer Not working/housewife	1 51 14 15 82	0.6 31.3 8.6 9.2 50.3
Monthly income <5000 SDG (<90.7 U.S. dollars) 5000–10,000 SDG (90.7–181.4 U.S. dollars) >10,000 and <20,000 SDG (>181.4 and <363 U.S. dollars) 20,000–30,000 SDG (363–544.5 U.S. dollars) >30,000 SDG (>544.5 U.S. dollars)	23 40 47 17 36	14.1 24.5 28.8 10.4 22.1
Living conditions With family With relative Alone	143 13 5	87.7 8 3.1
Loss of someone very close	43	26.4
Lack of social support	42	25.8
Smoking	6	3.7
Alcohol consumption	0	0

### 3.2. Clinical characteristics of the study participants

The majority of patients (91.4%) had type 2 DM, with 116 patients (71.8%) having been diagnosed for less than 10 years. Oral hypoglycemic agents were the primary treatment for the majority of the participants (64.4%). Complications from diabetes were present in 35.0% of the patients, with eye complications being the most common (26.4%). Over half (57.1%) had an HbA1c level greater than 7.0% in the past 3 months. Additionally, 38.7% had hypertension, while only 2.5% had a history of depression (Table 2).

**Table 2:** Clinical characteristics of the study participants and their self-care (N = 163).

Variables	Frequency	Percentage (%)
Clinical characteristics		
Type of diabetes mellitus Type 1 Type 2	14 149	8.6 91.4
Duration of diabetes mellitus (years) 1–10 >10	116 47	71.8 28.2
Diabetes mellitus therapy Insulin Oral hypoglycemic medication (OHM) Insulin and OHM	31 105 27	19 64.4 16.6
Presence of DM complications	57	35
Type of DM complications Eye complications Kidney complications Limbs complications Nerves complications Heart complication Other complications	43 2 13 1 5 0	26.4 1.2 8 0.6 3.1 0
HbA1c <6.5% 6.5%–7.0% >7.0%	22 27 93	13.5 16.6 57.1
Hypertension	63	38.7
History of depression	4	2.5
History of other psychiatric illnesses	0	0
Family history of any psychiatric illness	24	14.7

### 3.3. Prevalence of depression among the participants

**Figure 1.**
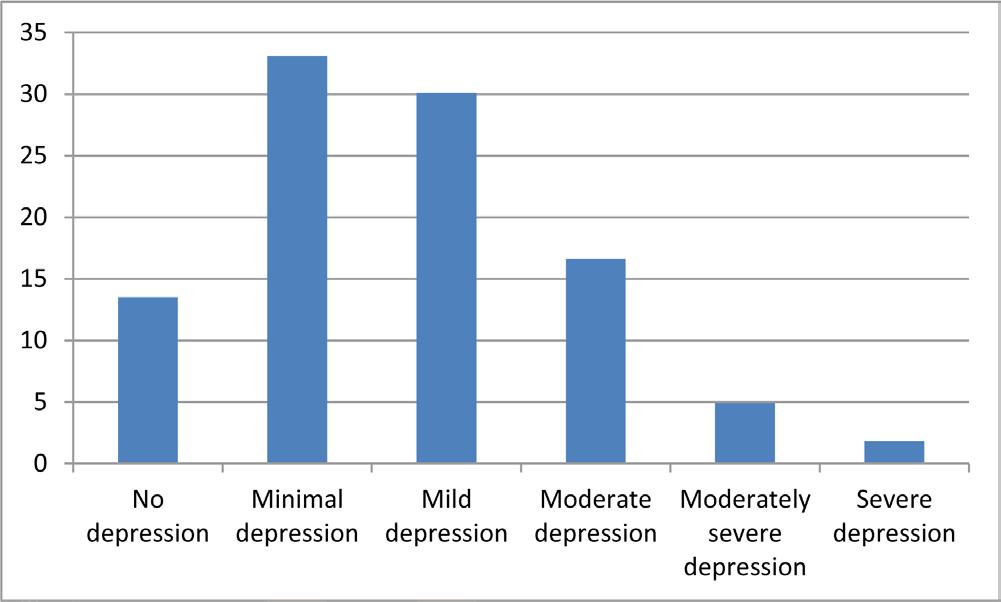
Prevalence of depression (%) among the participants (N = 163).

The overall prevalence of depression in this study was 86.5% (141 patients). Among the participants, 33.1% experienced minimal depression, 30.1% had mild depression, 16.6% had moderate depression, and only 1.8% suffered from severe depression (Fig. 1).

### 3.4. Diabetes self-care activities

Regarding diabetes self-care, 36.8% of participants either did not follow a healthy eating plan or did so only once per week. In terms of physical activity, 63.8% did not engage in specific exercise sessions or participated in only 1 session per week. Furthermore, 84.1% did not monitor their blood glucose levels in the last week, and 37.4% checked inside their shoes once or less per week (Table 3).

**Table 3:** Diabetes self-care activities (N = 163).

Diabetes self-care activities	Frequency	Percentage (%)
Diet Commitment to healthy diet during previous week: Once weekly or less 2–4 times per week 5–7 times per week Commitment to healthy diet during previous month: Once weekly or less 2–4 times per week 5–7 times per week	60 34 69 60 42 61	36.8 20.8 42.4 36.8 25.8 37.4
Exercise Commitment to practice exercise for at least 30 minutes during the previous week: Once weekly or less 2–4 times per week 5–7 times per week Commitment to specific exercise session during the previous week: Once weekly or less 2–4 times per week 5–7 times per week	34 29 100 104 28 31	20.9 17.8 61.4 63.8 17.2 19
Blood sugar testing Number of days in the previous week for testing the blood sugar: 1 day or less 2–4 days 5–7 days Number of days in the previous week for testing the blood sugar according to your doctor instructions: 1 day or less 2–4 days 5–7 days	137 23 3 141 18 4	84.1 14.2 1.8 86.5 11 2.5
Foot care Number of days in the previous week for checking your foot: 1 day or less 2–4 days 5–7 days Number of days in the previous week for checking your shoes for any harmful materials: 1 day or less 2–4 days 5–7 days	23 3 137 61 4 98	14.1 1.8 84 37.4 2.4 60.1

### 3.5. Association between depression and patients’ characteristics

The analysis revealed significant associations between depression and certain sociodemographic, individual, and social factors. Patients who experienced the loss of someone very close were more likely to have mild to severe depression (76.7%) than no depression to minimal depression (*P*-value = .001). Additionally, a lack of social support was significantly associated with depression status with higher percentages having mild to moderate depression (69%, *P*-value = .018). Other variables, such as age, gender, marital status, educational level, occupation, income, and smoking did not show significant associations with depression (Table 4).

**Table 4:** Univariate analysis of sociodemographic factor and depression status (N = 163).

Variable	No-minimal depression	Mild–severe depression	*P*-value
Age (years)	52 ± 20^a^	51 ± 25^a^	.338^b^
Gender Male Female	33 (55%) 43 (41.7%)	27 (45%) 60 (58.3%)	.100
Marital status Married Single Divorced Widow Separate	65 (49.6%) 2 (18.2%) 0 (0%) 9 (50%) 0 (0%)	66 (50.4%) 9 (81.8%) 1 (100%) 9 (50%) 2 (100%)	.110
Educational level Illiterate Informal education Primary education Secondary education University education Higher education	17 (48.6%) 5 (71.4%) 12 (33.3%) 22 (57.9%) 13 (35.1%) 7 (70%)	18 (51.4%) 2 (28.6%) 24 (66.7%) 16 (42.1%) 24 (64.9%) 3 (30%)	.065
Occupation Student Governmental employee Private sector employee Freelancer Not working/housewife	0 (0%) 25 (49%) 7 (50%) 7 (46.7%) 37 (45.1%)	1 (100%) 26 (51%) 7 (50%) 8 (53.3%) 45 (54.9%)	.890
Monthly income <5000 SDG (<90.7 U.S. dollars) 5000–10,000 SDG (90.7–181.4 U.S. dollars) >10,000 and <20,000 SDG (>181.4 and <363 U.S. dollars) 20,000–30,000 SDG (363–544.5 U.S. dollars) >30,000 SDG (>544.5 U.S. dollars)	14 (60.9%) 23 (57.5%) 17 (36.2%) 5 (29.4%) 17 (47.2%)	9 (39.1%) 17 (42.5%) 30 (63.8%) 12 (70.6%) 19 (52.8%)	.096
Living conditions With family With relative Dormitory Alone	71 (49.7%) 4 (30.8%) 0 (0%) 1 (50%)	72 (50.3%) 9 (69.2%) 5 (100%) 1 (50%)	.072
Loss of someone very close	10 (23.3%)	33 (76.7%)	.001**
Lack of social support	13 (31%)	29 (69%)	.018*
Smoking	4 (66.7%)	2 (33.3%)	.419
Alcohol consumption	0 (0%)	0 (0%)	-

a Median ± interquartile range.

b Calculated using Mann–Whitney test.

* *P*-value < .05.

** *P*-value < .01.

Regarding the association between depression and patients’ clinical characteristics, a significant association was found between depression and type of diabetes therapy, with patients on insulin experiencing the highest percentage of mild to severe depression (77.4%, *P*-value = .011). Additionally, family history of psychiatric illness was significantly correlated with depression (*P*-value = .001). Other clinical variables did not show significant associations with depression (Table 5).

**Table 5:** Association between depression and patients’ clinical factors (N = 163).

Variables	No-minimal depression	Mild–severe depression	*P*-value
Type of diabetes mellitus Type 1 Type 2	5 (35.7%) 71 (47.7%)	9 (64.3%) 78 (52.3%)	.392
Duration of diabetes mellitus (years) 1–10 >10	52 (44.8%) 24 (51.1%)	64 (55.2%) 23 (48.9%)	.47
Diabetes mellitus therapy Insulin Oral hypoglycemic medication Insulin and oral hypoglycemic medications	7 (22.6%) 55 (52.4%) 14 (51.9%)	24 (77.4%) 50 (47.6%) 13 (48.1%)	.011*
Presence of diabetes mellitus complications	21 (36.8%)	36 (63.2%)	.066
Types of diabetes mellitus complications Eye complications Kidney complications Limps complications Nerves complications Heart complications Other complications	16 (37.2%) 1 (50%) 6 (46.2%) 1 (100%) 2 (40%) 0 (0%)	27 (62.8%) 1 (50%) 7 (53.8%) 0 (0%) 3 (60%) 0 (0%)	.695
HbA1c <6.5 6.5–7.0 >7.0	13 (59.1%) 8 (29.6%) 47 (50.5%)	9 (40.9%) 19 (70.4%) 46 (49.5%)	.083
Hypertension	32 (50.8%)	31 (49.2%)	.397
History of depression	0 (0%)	4 (100%)	.124
History of other psychiatric illnesses	0 (0%)	0 (0%)	-
Family history of any psychiatric illness	4 (16.7%)	20 (83.3%)	.001**

* *P*-value < .05.

** *P*-value < .01.

### 3.6. Association between depression and diabetes self-care activities

The analysis showed a significant association between depression and diabetes self-care activities. Patients with mild to severe depression were significantly more likely to eat healthy only once per week or less (*P*-value < .001), engage in physical activity once weekly or less (*P*-value = .048) and committed to specific exercise session once weekly or less (*P*-value = .001). Moreover, blood sugar testing and regular checks for harmful materials in the shoes were significantly associated with depression status (*P*-value = .022 and .019, respectively). Participants who had mild to severe depression were mostly patients who checked their blood glucose and checked the inside of their shoes for 2 to 4 days per the previous week (78.3% and 75.0%, respectively) (Table 6).

**Table 6:** Association between depression and diabetes self-care activities (N = 163).

Diabetes self-care activities (days performed in the last week)	No-minimal depression	Mild–severe depression	*P*-value
Diet Commitment to healthy diet during previous week: Once weekly or less 2–4 times per week 5–7 times per week Commitment to healthy diet during previous month: Once weekly or less 2–4 times per week 5–7 times per week	16 (26.7%) 18 (52.9%) 42 (60.9%) 23 (38.3%) 17 (40.5%) 36 (59.0%)	44 (73.3%) 16 (47.1%) 27 (39.1%) 37 (61.7%) 25 (59.5%) 25 (41.0%)	.000** .048*
Exercise Commitment to practice exercise for at least 30 minutes during the previous week: Once weekly or less 2–4 times per week 5-7 times per week Commitment to specific exercise session during the previous week: Once weekly or less 2–4 times per week 5–7 times per week	6 (17.6%) 8 (27.6%) 62 (62.0%) 39 (37.5%) 14 (50.0%) 23 (74.2%)	28 (82.4%) 21 (72.4%) 38 (38.0%) 65 (62.5%) 14 (50.0%) 8 (25.8%)	.000** .001**
Blood sugar testing Number of days in the previous week for testing the blood sugar: 1 day or less 2–4 days 5–7 days Number of days in the previous week for testing the blood sugar according to your doctor instructions: 1 day or less 2–4 days 5–7 days	69 (50.4%) 5 (21.7%) 2 (66.7%) 70 (49.6%) 4 (22.2%) 2 (50.0%)	68 (49.6%) 18 (78.3%) 1 (33.3%) 71 (50.4%) 14 (77.8%) 2 (50.0%)	.022* .068
Foot care Number of days in the previous week for checking your foot: 1 day or less 2–4 days 5–7 days Number of days in the previous week for checking your shoes for any harmful materials: 1 day or less 2–4 days 5–7 days	7 (30.4%) 1 (33.3%) 68 (49.6%) 21 (34.4%) 1 (25.0%) 54 (55.1%)	16 (69.6%) 2 (66.7%) 69 (50.4%) 40 (65.6%) 3 (75.0%) 44 (44.9%)	.208 .019*

* *P*-value < .05.

** *P*-value < .01.

### 3.7. Regression analysis for factors affecting depression status

Binomial logistic regression was performed for factors affecting depression status (no depression to minimal depression vs. mild to moderate depression). The model was fit as indicated by Omnibus test (*P*-value < .001) with Nagelkerke R Square value of 0.550. Multiple variables were found to be significant. Loss of someone very close was significantly associated with 6.2 more prevalence of mild to severe depression than not losing them (odds ratio [OR] = 0.162, 95% confidence interval [CI] = 0.054–0.491, *P*-value = .001). Patients who are committed to 2 to 4 days of healthy diet per month had significantly almost 11.8 times more prevalence of mild to severe depression than having once or not having a healthy diet at all (OR = 11.833, 95% CI = 1.594–87.840, *P*-value = .016). However, when asking about the previous week's diet routines, commitment to a healthy diet 2 to 4 times per week was significantly associated with less mild to severe depression than patients having once or not having a healthy diet at all (OR = 0.118, 95% CI = 0.016–0.861, *P*-value = .035). Practicing at least 30 minutes of exercise for 5 to 7 times per week was significantly associated with fewer times of mild to severe depression than those who practice once or less per week (OR = 0.118, 95% CI = 0.028–0.503, *P*-value = .004). Moreover, commitment to 5 to 7 specific exercise session during the previous week was significantly associated with less times of mild to severe depression than those who practice once or less per week (OR = 0.259, 95% CI = 0.073–0.926, *P*-value = .038) (Table 7).

**Table 7:** Binomial logistic regression of factors affecting depression status (N = 163).

Variables	B	SE	*P*-value	Odds ratio	95% CI for odd ratio	
					Lower	Upper
Diabetes mellitus therapy (ref = insulin) Oral hypoglycemic medication Insulin and oral hypoglycemic medications	−1.003 −1.503	0.658 0.827	.181 .128 .069	0.367 0.222	0.101 0.044	1.333 1.125
Family history of any psychiatric illness (ref = Yes) No	−1.350	0.692	.051	0.259	0.067	1.006
Loss of someone very close (ref = Yes) No	−1.818	0.565	.001**	0.162	0.054	0.491
Availability of social support (ref = Yes) No	0.349	0.632	.581	1.417	0.411	4.893
Commitment to healthy diet during previous week (ref = Once weekly or less) 2–4 times per week 5–7 times per week	−2.136 −1.340	1.014 1.031	.101 .035* .194	0.118 0.262	0.016 0.035	0.861 1.976
Commitment to healthy diet during previous month (ref = Once weekly or less) 2–4 times per week 5–7 times per week	2.471 0.772	1.023 1.017	.016* .016* .448	11.833 2.165	1.594 0.295	87.840 15.903
Commitment to practice exercise for at least 30 minutes during the previous week (ref = Once weekly or less) 2–4 times per week 5–7 times per week	−0.155 −2.135	0.800 0.739	.001** .846 .004**	0.856 0.118	0.178 0.028	4.110 0.503
Commitment to specific exercise session during the previous week (ref = Once weekly or less) 2–4 days 5–7 days	−0.350 −1.350	0.648 0.649	.115 .589 .038*	0.705 0.259	0.198 0.073	2.511 0.926
Number of days in the previous week for testing the blood sugar (ref = 1 day or less) 2–4 days 5–7 days	1.094 −0.448	0.733 1.365	.306 .135 .743	2.986 0.639	0.711 0.044	12.551 9.278
Number of days in the previous week for checking your shoes for any harmful materials (ref = 1 day or less) 2–4 days 5–7 days	0.248 −0.616	1.442 0.513	.474 .863 .230	1.282 0.540	0.076 0.197	21.642 1.476
Constant	5.520	1.297	.000	249.666		

CI = confidence interval, SE = standard error.

* *P*-value < .05.

** *P*-value < .01.

## 4. Discussion

This study aimed to determine the prevalence of depression among diabetic patients in Khartoum State, besides exploring its association with various patient factors. We found a high overall prevalence of depression among the patients, primarily of minimal and mild severity. This rate is 3 times higher than that reported in the USA between 2011 and 2019.^[15]^ However, it is similar to the findings from a previous study in Tanzania, which used the same depression assessment tool.^[16]^ This highlights the disparity in depression rates between developing and developed countries. These differences are likely due to variations in socioeconomic factors, healthcare access, and cultural attitudes towards mental health.

Regarding diabetes control, 57% of our participants exhibited poor glycemic control, indicated by HbA1c levels above 7%. This finding reflects an improvement compared to 2 previous Sudanese studies, in which over 80% of patients with type 1 and type 2 diabetes had inadequate glycemic control.^[17,18]^ This difference could be attributed to our data being collected from hospital patients who were under regular follow-up.

Our findings highlighted notable discrepancy in both physical activity and blood glucose monitoring among patients. While most participants engaged in at least 30 minutes of exercise 5 to 7 days in the past week, only a small percentage adhered to blood glucose monitoring in the last week. This discrepancy underscores the challenges in maintaining consistent self-care practices essential for diabetes management, including both exercise and blood glucose monitoring. These findings are consistent with studies conducted in the USA and Ghana,^[19,20]^ indicating that barriers to self-care adherence are common across diverse populations.

There is a significant association between a lack of social support and an increased risk of depression in diabetic patients, underscoring the critical role that emotional and social networks play in managing chronic conditions. Social isolation can exacerbate feelings of helplessness and stress, making it harder for patients to cope with the demands of diabetes, which may explain this relationship. These findings are consistent with previous research,^[21]^ further reinforcing the need for integrating psychosocial support into diabetes care. Friendship support connected to diabetes consistently contributed more than family support related to diabetes. According to these results, assistance from outside the family was more crucial for adjusting to DM and has been shown to have direct and buffering effects in the mitigation of depressive symptoms.^[22]^

The loss of someone close was, also, significantly associated with depression in diabetic patients, as grief can intensify emotional distress and interfere with adherence to treatment and self-care activities.^[23]^ This aligns with findings from a study conducted in Ethiopia.^[24]^ This type of loss can result in extended periods of sadness and withdrawal from essential health routines, further increasing the risk of depression.

In this study, we found a statistically significant correlation between depression and the type of diabetic medications used, with insulin usage being associated with higher levels of depression. This matches what is described in the literature about users of specific antidiabetic drugs having a lower risk of depression compared to nonusers.^[25,26]^ Treatment with insulin has been associated with an increased risk of depression. Due to reduced levels of endogenous insulin, individuals receiving insulin therapy were shown to be more vulnerable to metabolic dysregulation than those with residual insulin secretory activity.^[26]^

Our study indicated that the presence of diabetes complications may increase the likelihood of experiencing mild to severe depression; however, this association did not reach statistical significance. This finding aligns with previous studies highlighting the link between depression and diabetes-related complications,^[27]^ reinforcing the importance of addressing mental health to improve long-term outcomes in diabetic patients.^[28]^

Patients with a family history of psychiatric illness showed a significant association with depression, this was also described in a study from Sudan.^[29]^ This might be explained by the hypothesis suggesting that these results are the product of familial aggregation between type 2 DM and major psychiatric disorders and can be further justified by a shared genetic pool as well as certain environmental factors.^[30]^ Studies have supported this assumption by demonstrating a significant genetic overlap between type 2 DM and depression.^[31]^ These patients should have their depressive symptoms regularly monitored and be provided with psychological and educational interventions.

A strong association was found between mild–severe depression and infrequent diabetes self-care activities (once weekly or less). This was consistent with a previous study conducted in the USA using the same Self-Reported Diabetes Self-Care Activities Questionnaire scale, which found that patients with major depression were more likely to neglect self-care compared to nondepressed patients, with the exception of the frequency of blood glucose monitoring and foot checks.^[19]^ The lack of consistent self-care can lead to serious complications, including blindness, heart failure, and renal failure in diabetic patients.

This study is one of the few to assess the prevalence of depression among Sudanese diabetic patients. The findings provide important data that can guide targeted interventions and serve as a reference for future research. However, the study has some limitations. The sample size is relatively small, and there is no control group of nondiabetic individuals for comparison. Additionally, the potential use of medications with depression-inducing effects among participants was not considered, which could have influenced the results.

## 5. Conclusion

The prevalence of depression among diabetic patients in this study was 86.5%, with the majority experiencing minimal to mild depression. Depression in diabetic patients was significantly associated with diabetes self-care activities, type of diabetes medications, family history of psychiatric illness, loss of a close person, and lack of social support. To improve clinical outcomes for diabetic patients with comorbid depression, clear healthcare pathways utilizing a multidisciplinary team approach are essential. Further research with larger sample sizes and the inclusion of nondiabetic control groups is necessary. Additionally, community-based studies investigating the prevalence of depression and its associated factors among diabetics are recommended.

## Acknowledgments

We are deeply grateful to all the participants who dedicated their time to this study. Their involvement was essential to its success.

## Author contributions

**Conceptualization:** Sara Emad.

**Data curation:** Sara Emad, Sara Elawad, Mohamed H. Elbadawi, Leena Khalid, Tibyan Yahya Mohammedelhassan Abdalhamed, Wafa Sosal, Aya M. Haiba.

**Formal analysis:** Sara Emad, Mohamed H. Elbadawi.

**Investigation:** Sara Emad, Sara Elawad, Shaima Omer Moahmed Elawad, Ahmed Balla M. Ahmed, Mohamed H. Elbadawi, Leena Khalid, Tibyan Yahya Mohammedelhassan Abdalhamed, Wafa Sosal, Aya M. Haiba.

**Methodology:** Sara Emad.

**Validation:** Sara Emad.

**Writing – original draft:** Sara Emad, Sara Elawad, Shaima Omer Moahmed Elawad, Ahmed Balla M. Ahmed, Mohamed H. Elbadawi, Leena Khalid, Tibyan Yahya Mohammedelhassan Abdalhamed, Wafa Sosal, Aya M. Haiba.

**Writing – review & editing:** Sara Emad, Shaima Omer Moahmed Elawad, Ahmed Balla M. Ahmed, Mohamed H. Elbadawi.
